# Supplementary material for: Prevalence and Outcomes Associated with Vitamin D Deficiency among Indexed Hospitalizations with Cardiovascular Disease and Cerebrovascular Disorder—A Nationwide Study
Source: Medicines (Basel). 2020 Nov 22;7(11):72. doi: 10.3390/medicines7110072 (PMC7700427; doi:10.3390/medicines7110072)
Supplement: Supplementary file 1 [file medicines-07-00072-s001.pdf]

Supplementary Materials: Prevalence and Outcomes Associated with Vitamin D Deficiency among Indexed Hospitalizations with Cardiovascular Disease and Cerebrovascular Disorder—A Nationwide Study

Urvish Patel, Salma Yousuf, Komal Lakhani, Payu Raval, Nirmaljot Kaur, Toochukwu Okafor, Chail Shah, Harmandeep Singh, Mehwish Martin, Chika Nwodika, Angelina Yogarajah, Jigisha Rakholiya, Maitree Patel, Raja Chandra Chakinala and Shamik Shah

Table S1. ICD-10 CM codes.

Intracerebral Hemorrahge	I61.xx
Subarachnoid Hemorrhage	I60.xx
Ischemic Heart disease	I20.xx-I25.xx
(Atrial fibrillation	I48
Acute MI	I21.xx
Angina	I20.xx
Hypertension	I10.xx-I16.xx
Diabetes mellitus	E08.xx-E13.xx
Lipid disorder	E78.xx
Smoking	F17.xx
Obesity	E66.xx
Renal Dysfunction	N18.xx
Alcohol related disorder	F10.xx
Drug Abuse	F11.xx-F19.xx
AIDS	B20, R75, Z21

Table S2. Elixhauser comorbidities—coding algorithms.

- Congestive heart failure: I09.9, I11.0, I13.0, I13.2, I25.5, I42.0, 142.5-I42.9, I43.x, I50.x, P29.0
- Cardiac arrhythmias: I44.1-I44.3, I45.6, I45.9, I47.x-I49.x, ROO.O, ROO.1, ROO.8, T82.1, Z45.0, Z95.0
- Valvular disease: A52.0, I05.x-I08.x, I09.1, I09.8, I34.x-I39.x, Q23.O- Q23.3, Z95.2, Z95.4
- Pulmonary circulation Disorders: I26.x, I27.x, I28.0, I28.8, I28.9
- Peripheral vascular disorders: I70.x, I71.x, I73.1, I73.8, I73.9, I77.1, I79.0, I79.2, K55.1, K55.8, K55.9, Z95.8, Z95.9
- Hypertension, uncomplicated: I10.x
- Hypertension, complicated: I11.x-I13.x, I15.x

- Paralysis: G04.1, G11.4, G80.1, G80.2, G81.x, G82.x, G83.0-G83.4, G83.9
- Other neurological disorders: G10.x-G 13.x, G20.x- G22.x, G25.4, G25.5, G31.2, G31.8, G31.9, G32.x, G35.x-G37.x, G40.x, G41.x, G93.1, G93.4, R47.0, R56.x
- Chronic pulmonary disease: I27.8, 127.9, J40.x-J47.x, J60.x-J67.x, J68.4, J70.1, J70.3
- Diabetes, uncomplicated: E10.0, E10.1, E10.9, E11.0, E11.1, E11.9, E12.0, E12.1, E12.9, E13.0, E13.1, E13.9, E14.0, E14.1, E14.9
- Diabetes, complicated: E10.2-E10.8, E11.2-E11.8, E12.2-E12.8, E13.2-E13.8, E14.2-E14.8
- Hypothyroidism: E00.x-E03.x, E89.0
- Renal failure: I12.0, I13.1, N18.x, NI9.x, N25.0, Z49.0-Z49.2, Z94.0, Z199.2
- Liver disease: B18.x, I85.x, I86.4, I98.2, K70.x, K71.1, K71.3- K71.5, K71.7, K72.x- K74.x, K76.0, K76.2- K76.9. Z94.4
- Peptic ulcer disease excluding bleeding: K25.7, K25.9, K26.7, K26.9, K27.7, K27.9, K28.7, K28.9
- AIDS/HIV: B20.x-B22.x, B24.x
- Lymphoma: C81.x-C85.x, C88.x, C96.x, C90.0, C90.2
- Metastatic cancer: C77.x-C80.x
- Solid tumor without metastasis: C00.x-C26.x, C30.x-C34.x, C37.x-C41.x, C43.x, C45.x-C58.x, C60.x-C76.x, C97.x
- Rheumatoid arthritis/ collagen vascular diseases: L94.0, L94.1, L94.3, M05.x, M06.x, M08.x, M12.0, M12.3, M30.x, M31.0-M31.3, M32.x-M35.x, M45.x, M46.1, M46.8, M46.9
- Coagulopathy: D65-D68.x, D69.1, D69.3-D69.6
- Obesity: E66.x
- Weight loss: E40.x-E46.x, R63.4, R64
- Fluid and electrolyte disorders: E22.2, E86.x, E87.x
- Blood loss anemia: D50.0
- Deficiency anemia: D50.8, D50.9, D51.x-D53.x

- Alcohol abuse: F10, E52, G62.1, I42.6, K29.2, K70.0, K70.3, K70.9, T51.x, Z50.2, Z71.4, Z72.1
- Drug abuse: F11.x-F16.x, F18.x, F19.x, Z71.5. Z72.2
- Psychoses: F20.x, F22.x-F25.x, F28.x, F29.x, F30.2, F31.2, F31.5
- Depression: F20.4, F31.3-F31.5, F32.x, F33.x, F34.1, F41.2, F43.2

Source: Quan H, Sundararajan V, Halfon P, et al. Coding algorithms for defining Comorbidities in ICD-9-CM and ICD-10 administrative data. Med Care. 2005 Nov; 43(11): 1130-9.
